# *Buyang Huanwu* Decoction enhances hippocampal-cortical connectivity remodeling via sonic hedgehog signaling to ameliorate memory dysfunction in cerebral ischemic rats

**DOI:** 10.1186/s13020-025-01122-0

**Published:** 2025-09-19

**Authors:** Yun Lu, Ziyue Lin, Hanyu Wang, Yuming Zhuang, Jingting Jia, Yuxuan Wang, Le Yang, Manzhong Li, Mingcong Li, Binbin Nie, Rui Zhang, Xu Pan, Jianfeng Lei, Haiyan Zou, Hui Zhao

**Affiliations:** 1 Department of Chinese Pharmacy, Beijing Jishuitan Hospital, Capital Medical University, Beijing, 100035 China; 2 School of Traditional Chinese Medicine, Capital Medical University, #10, Youanmenwai Xitoutiao, Fengtai District, Beijing, 100069 China; 3 Department of Pharmacy, The Second Affiliated Hospital of Dalian Medical University, Dalian, 116023 China; 4 Department of Pharmacy, Beijing Shijitan Hospital, Capital Medical University, Beijing, 100038 China; 5 Beijing Engineering Research Center of Radiographic Techniques and Equipment, Institute of High Energy Physics, Chinese Academy of Sciences, Beijing, 100049 China; 6 Medical Imaging Laboratory of Core Facility Center, Capital Medical University, Beijing, 100069 China

**Keywords:** *Buyang Huanwu* Decoction, Post-stroke memory dysfunction, Hippocampal-cortical connectivity, Neurogenesis, Sonic hedgehog signaling pathway

## Abstract

**Background:**

Although *Buyang Huanwu* Decoction (BHD) has been shown to promote functional recovery of memory following ischemic stroke, the precise mechanisms underlying its therapeutic effects remain incompletely understood. This study aimed to investigate the impact of BHD on hippocampal-cortical connectivity and elucidate the associated neurobiological mechanisms mediating its restorative effects.

**Methods:**

A permanent middle cerebral artery occlusion (MCAO) rat model was established to simulate ischemic stroke conditions for subsequent experimental analyses. MCAO rats received daily intragastric administration of BHD over a 30-day treatment period. Cognitive performance, specifically spatial learning and memory, was assessed using the Morris water maze (MWM) test. Structural alterations in the hippocampus and cortex were quantified through magnetic resonance imaging (MRI), while functional neuronal activity was evaluated using blood-oxygen-level-dependent (BOLD) imaging, including amplitude of low-frequency fluctuation (ALFF) and regional homogeneity (ReHo) analyses. Seed-based functional connectivity analysis derived from BOLD signals was employed to investigate dynamic changes in hippocampocortical connectivity. Additionally, the involvement of the Sonic hedgehog (Shh) signaling pathway was examined using Western blotting to elucidate potential molecular mechanisms underlying the therapeutic effects of BHD.

**Results:**

Therapeutic administration of BHD significantly ameliorated ischemia-induced memory impairments, attenuated structural damage in hippocampal and cortical regions, and restored neuronal activity levels in the post-stroke hippocampal regions. Notably, BHD treatment promoted functional reorganization of hippocampal-cortical connectivity, concomitant with the modulation of the Shh signaling pathway in both hippocampal and cortical regions.

**Conclusions:**

The treatment with BHD facilitated the remodeling of the connectivity between the hippocampus and cortex, and ultimately alleviated memory dysfunction following stroke. These findings hold great promise in promoting the development of BHD research and enhancing its clinical utility.

**Supplementary Information:**

The online version contains supplementary material available at 10.1186/s13020-025-01122-0.

## Background

Globally, stroke ranks as the leading cause of adult disability [1]. Ischemic stroke-induced memory dysfunction has been shown to elevate the risk of dementia [2]. Currently, traditional Chinese medicinal preparations have drawn significant attention in the treatment with cerebral ischemia [3–5]. Buyang Huanwu Decoction (BHD) has been reported to enhance neurological function following stroke in clinical practice [3, 6]. BHD, a classic Traditional Chinese Medicine formula documented in Qing-ren Wang’s *Yilin Gaicuo* (*Correction of Errors in Medical Classics*), is clinically prescribed for post-stroke recovery, specifically targeting Qi deficiency and blood stasis syndrome [7–9]. Its therapeutic effects are achieved through a combination of strongly tonifying Qi and activating blood circulation to restore the flow of Qi and blood in the meridians. Pharmacological studies have demonstrated that bioactive constituents of BHD exhibit detectable distribution in cerebral tissues and substantiated its efficacy in promoting neurogenesis and facilitating the structural and functional reorganization of the neurovascular unit [10–13]. Previous investigations have consistently demonstrated that BHD facilitates memory function recovery through the modulation of hippocampal neuroplasticity, as evidenced by its ability to enhance synaptic reorganization and neural circuit remodeling [5, 14]. Nevertheless, the neuronal mechanism of BHD treatment on the post-stroke memory deficits remains still unclear.

It is important to emphasize that neuronal function hinges on inter-regional information communication, particularly in the context of learning and memory functions [15]. Of note, a growing body of evidence suggests that the hippocampus plays a pivotal role in the execution of memory functions [15–19]. Moreover, several cortical regions, such as the entorhinal cortex, have been reported to have bidirectional neuronal projections with hippocampus [16, 20]. Additionally, the cingulate cortex and retrosplenial cortex also receive major projections from the hippocampus and are involved in the memory process [16, 21]. Cerebral ischemic damage has been shown to impede the regional information communication between the hippocampus and the cortex, ultimately resulting in memory dysfunction [22, 23]. However, to date, no research has been conducted on how BHD impacts the information flow between the hippocampus and the cortex following an ischemic stroke.

Functional Magnetic resonance imaging (fMRI) has been proven effective and is widely employed to detect regional functional communication. Blood-oxygen-level-dependent (BOLD) imaging, a prevalent fMRI technique, was utilized to evaluate the level of neuronal activity via detecting blood oxygen changes [24, 25]. The amplitude of low-frequency fluctuations (ALFF) based on BOLD represents the level of regional spontaneous activity, and regional homogeneity (ReHo) quantifies the homogeneity of neuronal activity [26, 27]. Seed-based functional connectivity (FC) analysis was the popular method for assessing the changes of inter-regional functional connectivity [27, 28]. Previous findings have indicated that focal cerebral ischemia disturbed the hippocampal-cortical connectivity [23]. Nevertheless, the impact of BHD on the connectivity within hippocampal-cortical circuits remains unclear.

The canonical Sonic Hedgehog (Shh) signaling pathway is critically involved in the structural and functional reorganization of hippocampal neuronal networks [29]. The molecular cascade of Shh signaling initiates with the binding of Shh ligand to its transmembrane receptor, Patched (Ptch), which subsequently relieves the inhibition of Smoothened (Smo), a G-protein-coupled receptor (GPCR)-like protein. This activation triggers downstream signaling events culminating in the nuclear translocation and activation of the transcription factor Gli1, which orchestrates the expression of genes essential for neuronal plasticity and network remodeling. Accumulating evidence has demonstrated the pivotal role of Shh signaling in mediating hippocampal neurogenesis and synaptic reorganization following ischemic stroke [29]. However, the precise mechanisms by which Shh signaling modulates hippocampocortical circuit remodeling, particularly in the context of post-stroke treatment with BHD, remain to be fully elucidated. Further investigation is required to delineate the specific molecular and cellular pathways through which Shh signaling contributes to BHD-induced neural repair and functional recovery.

Herein, we aimed to use BOLD-based functional connectivity analysis to ascertain alterations in hippocampal-cortical connectivity following BHD treatment. Particular emphasis was placed on the remodeling of hippocampal-cortical connectivity by activating the Shh signaling pathway. The study will supplement the comprehension of the mechanisms underlying BHD treatment promoting memory remodeling after stroke.

## Methods

### Animals

Forty-five 8-week-old adult male SPF Sprague–Dawley rats (300–320 g) were procured from Vital River Laboratory Animal Technology Co. Ltd.. The rats were accommodated within the secure and environmentally controlled facilities at Capital Medical University (SYXK [jing] 2018–0030), where a 12-h light/dark cycle was maintained, and the temperature was regulated. The operational procedure was carried out in strict compliance with the regulations set forth by the Animal Ethics Committee of Capital Medical University (Permit Number: AEEI-2022–072).

### MCAO model and experimental design

Rats were anesthetized with the 5% isoflurane and maintained with 2% isoflurane in an oxygen-air mixture (1:1). To establish the cerebral ischemic model, a permanent occlusion of the right middle cerebral artery (MCA) was implemented, following the method described previously [30]. The vital signs of the rats were continuously monitored via a rat monitoring system (Small Animal Instruments Inc., USA). A warm water circulation system was employed to keep the rats’ body temperature at 37 °C.

The exclusion criteria comprised (1) absence of circling and walking towards the opposite side, and (2) a lack of notable tissue damage in T2WI maps of rats, which was earlier reported [31, 32]. A total of 25 rats underwent MCAO modeling. Four rats died within the first 3 days post-modeling. Three rats showing no behavioral deficits during the 24-h post-modeling neurobehavioral assessment were excluded. Two rats with no apparent tissue damage on T2WI were excluded from statistical analysis. The remaining 16 rats were randomly allocated to the MCAO + Saline (MS) group (n = 8) and MCAO + BHD (MB) group (n = 8). 16 rats without occlusion operation were recruited to the Sham + Saline (SS) group and Sham + BHD (SB) group.

### BHD preparation and treatment

The components of BHD were displayed in the Table 1. The name of the components was checked by http://www.theplantlist.org. The Chinese materia medica used in this study were purchased from the Beijing Tong Ren Tang Co. Ltd (Beijing, China). Our previous study has documented the preparation procedures and reported the chemical fingerprint of the extract tested of BHD via HPLC [14].

**Table 1:** The components of BHD

Latin name	Amount	Medical Part
*Astragalus mongholicus* Bunge	120 g	Root
*Angelica sinensis* (Oliv.) Diels	6 g	Root
*Paeonia lactiflora* Pall	4.5 g	Root
*Carthamus tinctorius* L	3 g	Flower
*Prunus persica* (L.) Batsch	3 g	Seed
*Ligusticum chuanxiong* Hort	3 g	Root
*Pheretima aspergillum* (E. Perrier)	3 g	Body

The intragastrical administration of BHD (16.6 g/kg) to the SB and MB group was performed from 24 h after MCAO for continuous 30 days. Normal saline (10 mL/kg) was intragastrically administered to the MS and SS group. The dose of BHD was as sevenfold as that of a person weighing 60 kg, which was optimized in our previous study [33–35].

### Magnetic resonance imaging protocols

5% Isoflurane vaporized in an oxygen-air mixture (1:1) was used for the anesthetization of rats. Dexmedetomidine (0.015 mg/kg) was intramuscularly injected to rats. After totally anesthetized, the rats were placed in the bed of the MRI machine with 2% isoflurane to maintain anesthetization. When the fMRI processing, 0.20%–0.25% isoflurane was used to remain the breathing rate of rats ranged from 60 to 85 breaths per minute. The vital signs and temperature of the rats were monitored and maintained as described previously.

On the 31st day following the surgery, we used a 7.0 Tesla PharmaScan imaging device (Bruker, Germany) to perform MRI scanning, including T2WI, Diffusion tensor imaging (DTI), and BOLD scanning, as reported previously [31, 36]. The parameters of these sequences were shown in the *Supplementary material*. ImageJ was utilized to obtain the infarct volume through plus every volume of individual slice of T2 maps [32]. The maps of and DTI were reconstructed with software utilized was Paravision version 5.1 (Bruker, Germany) to calculate the value of fraction anisotropy (FA) of the hippocampus (Hip), cingulate cortex (Cg), retrosplenial cortex (RSC) and entorhinal cortex (Ent) [37].

### ALFF and ReHo maps generation

BOLD image was processed via SPM-derived spmratIHEP and REST, as reported previously [36, 38]. BOLD images were processed using a self-adaptive template, with intracranial tissues segmented to create a binary mask. Preprocessing included slice-timing correction, motion realignment, spatial normalization to a standard rat brain atlas, Gaussian smoothing (FWHM = 1 mm), detrending, and bandpass filtering (0.01–0.1 Hz). Based on the BOLD-derived images, each voxel’s power spectrum was computed using a rapid Fourier transform, whose square root was obtained to gain the maps of the amplitude of low frequency fluctuation (ALFF). Moreover, Kendall’s coefficient of the preprocessed images was obtained through the concordance between the time series of a given voxel and that of the nearest 26 voxels, which constituted to the maps of regional homogeneity (ReHo).

### Functional connectivity maps generation

Seeds were located respectively at the right and left hippocampus, which was based on the regions of the BHD-induced raised FA value. The images of functional connectivity were generated by calculating the Pearson’s correlation between the time series of each voxel in the seed and those of each voxel in the brain.

### Morris water maze test

Morris water maze (MWM) experiment involved the hidden-platform experiment, the probe experiment and the switched-platform experiment. The operation of MWM experiment was previously reported [23]. The hidden-platform was located in the quadrant II during the hidden-platform test. A video analysis software (Jiliang, China) was used. The hidden-platform experiment measured the length of escape path required to locate the concealed platform. The Probe experiment detected the percentage of path in the quadrant II to find the site of the hidden-platform. The switched-platform experiment detected the length of the escape path to locate the switched-platform in the other quadrants.

### Tissue Processing

The details of tissue Processing was reported previously [23]. The coronal sections between Bregma – 2.2 to – 3.0 mm of 4 random rats per group were prepared for immunofluorescence staining. The tissue of the hippocampus and cortical areas of the remanent 4 rats per group was remained for Western Blotting.

### Immunofluorescence staining analysis

Double-label immunofluorescence staining's operation has been reported before [32]. The primary antibodies used in the experiment involved anti-Ki67 (Abcam, ab16667, 1:100), anti-GFAP (Millipore, MAB360, 1:600), anti-MAP-2 (Abcam, ab32454,1:200). The secondary antibodies conjugated with Alexa Fluor 488 (SouthernBiotech, 1036–02, 1:400) and 594 (SouthernBiotech, 4030–03, 1:400) were used. Three randomly selected, non-overlapping regions of the peri-infarct cortex were obtained using fluorescent microscopy in order to count the number of Ki67^+^ , Ki67/MAP-2^+^ , and Ki67/GFAP^+^ cells. Normalization of the data was done using the number of positive cells/mm^2^. Data acquisitions and analysis were performed under blinded experimental conditions.

#### Western Blotting Analysis

The operation of Western Blotting was earlier described [32]. The primary antibodies used in Western Blotting involved GLI1 (Proteintech, 66905–1-Ig, 1:20,000), SMO (Proteintech, 20787-1-AP, 1:20,000), SHH (Proteintech, 20697-1-AP, 1:10,000), Cyclin D1 (GeneTex, GTX108624, 1:10,000), Nestin (GeneTex, GTX630201, 1:10,000), Ptch1 (GeneTex, GTX64432, 1:5000), GAPDH (GeneTex, GTX100118, 1:40,000), β-tubulin (MICS, 1879-1, 1:40,000). ChemiDoc XRS + Imaging System (Bio-Rad, USA) was used to visualize the protein band. ImageJ was used to acquire the intensity of protein band. Data was presented as the relative intensity which was normalized to the intensity of GAPDH.

#### Statistical Analysis

Matlab 2014 and SPSS 26.0 were used for statistical analysis. The data's normality was evaluated using the Shapiro–Wilk test. For the data with a normal distribution, the two-way ANOVA was used to analyze the inter-group differences. The Bonferroni test was then used as a post hoc analysis. The correlation coefficient between FC values and behavior parameters was examined using Pearson's correlation test. At *P* < 0.05, differences were deemed statistically significant.

One-way ANOVA was performed to locate the inter-group significant differences in the ALFF, ReHo and FC maps. *Post-hoc* two-sample t test was performed to reveal the distribution of inter-group significant differences in ALFF, ReHo and FC maps [39]. Gaussian random field (GRF) theory was used to correct for results (the level of voxel, *P* < 0.005; the level of cluster, cluster size > 50).

## Results

### BHD ameliorated cerebral ischemic injury and promoted memory function remodeling in the MCAO rats

**Fig. 1:**
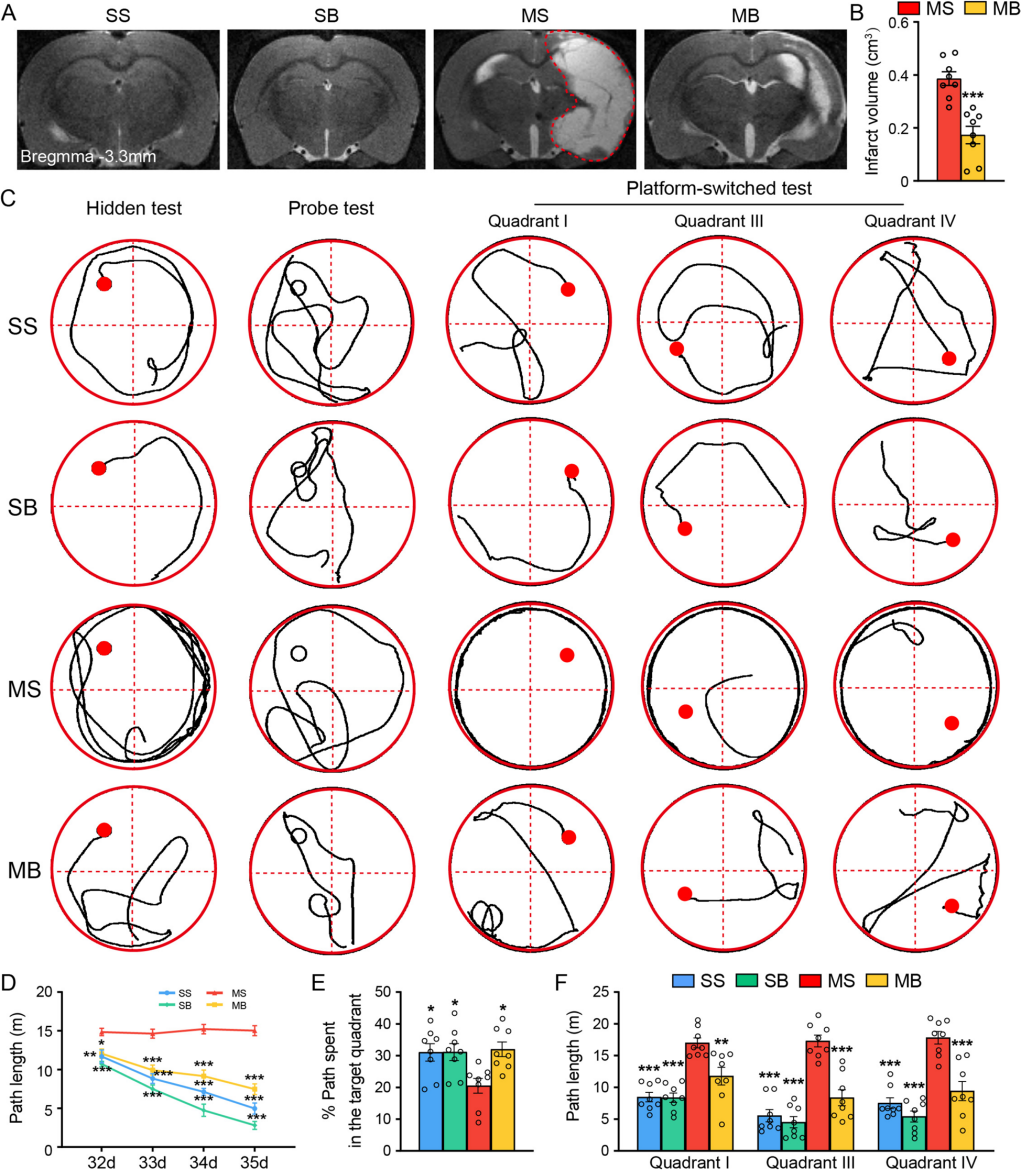
BHD ameliorated cerebral ischemic lesion and promoted the remodeling of the memory function in MCAO rats. **A** Representative T2WI images. Infarct area was demarcated by red dotted lines. **B** The quantitative results of the infarct volume. **C** Represent trajectories of the hidden-platform experiment, probe experiment and platform-switched experiment. The quantitative results of **D** the path length in the hidden-platform experiment, **E** the percentage of path in the quadrant II in the probe experiment, and **F** the path length in the switched-platform experiment. Mean ± SEM, *N* = 8. ^*^P < 0.05, ^**^P < 0.01, ^***^P < 0.001 vs. the MS group

T2WI images showed abnormally increased signal in the region of the right MCA (Fig. 1A). BHD significantly decreased the infarct volume of rats after ischemic stroke (Fig. 1B).

The results of the hidden-platform experiment showed a significant time × BHD interaction on the escape path identified with the two-way ANOVA. From day 32 to day 35, the path length of MS group increased relative to that of SS group. (Fig. 1C, D). BHD treatment significantly decreased the escape path of the rats following cerebral ischemia from 32nd to 35th day (Fig. 1C, D).

The results of the probe experiment identified the less path of MS group spent in the quadrant II compared to that of SS group (Fig. 1C, E). BHD treatment raised the path of rats with MCAO swimming in the quadrant II (Fig. 1C, E).

The results of the platform-switched experiment showed that the pathways to the switched platform were longer for the MS group in quadrants I, III, and IV than for the SS group. (Fig. 1C, F). BHD treatment decreased the path to locate the switched-platform following cerebral ischemia (Fig. 1C, F).

### BHD decreased microstructural lesion of the cortex and hippocampus in the MCAO rats

**Fig. 2:**
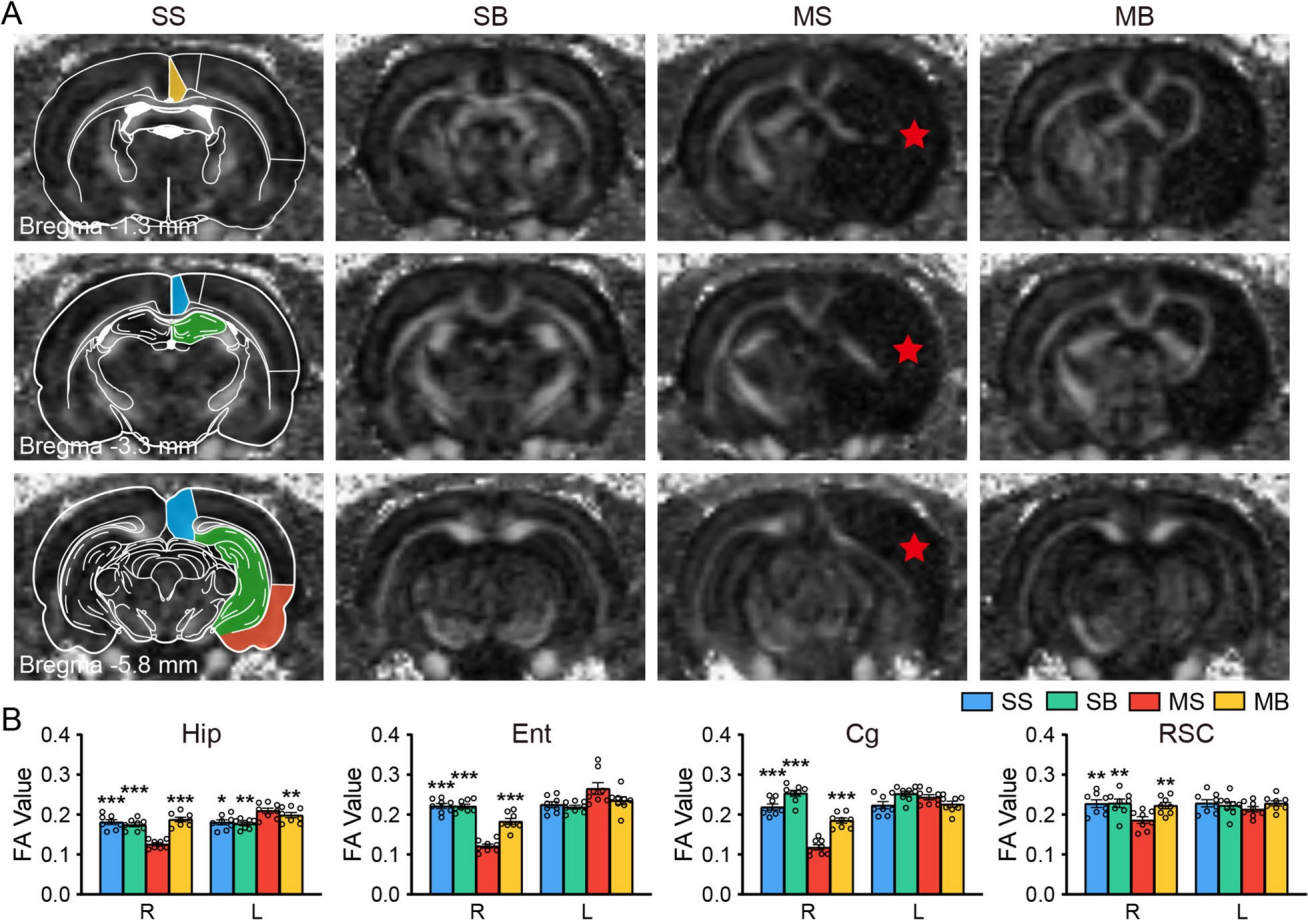
BHD ameliorated the micro-structural lesion of the hippocampus and cortex after stroke. **A** Typical FA maps. Schematic diagram in the Paxinos space. The infarct region was labelled with red star. **B** The quantitative FA results. Mean ± SEM, N = 8. ^*^P < 0.05, ^**^P < 0.01, ^***^P < 0.001 vs. the MS group

According to the FA results, the right hippocampus, entorhinal cortex, cingulate cortex, and retrosplenial cortex showed lower FA values in the MS group than in the SS group. (Fig. 2). Meanwhile, MS group displayed the increased FA value in the left hippocampus. MB group showed the reversed FA values in the corresponding regions compared to MS group (Fig. 2).

### BHD improved regional neuronal activity of the hippocampus in the MCAO rats

**Fig. 3:**
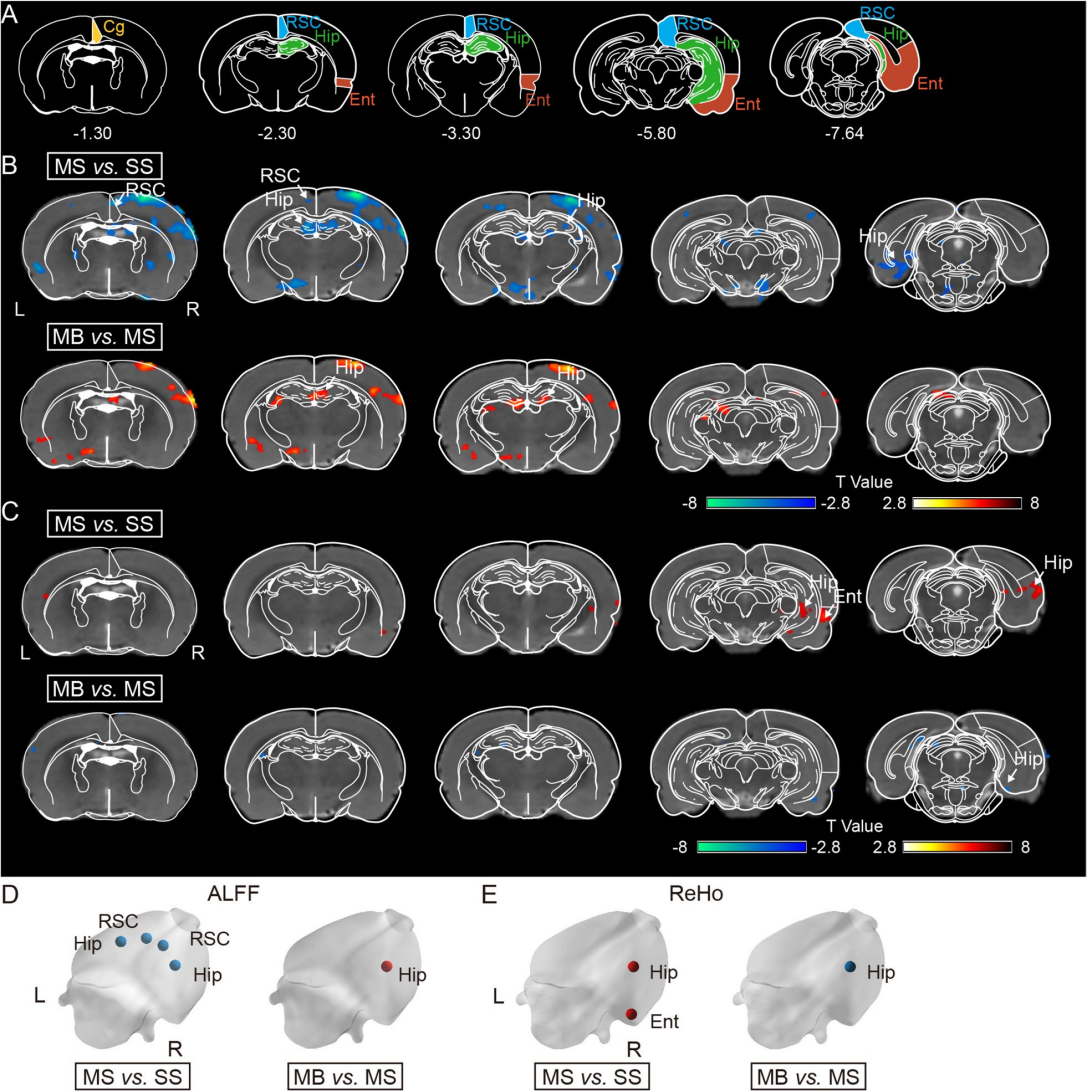
BHD improved regional spontaneous activity and regional homogeneity of hippocampus and cortex after cerebral ischemia. **A** Schematic illustration in the Paxinos space. The distribution of significant differences of **B** ALFF and **C** ReHo (FDR corrected, *P* < 0.005). The color overlay denotes the magnitude of group differences, with warm/cold hues representing higher/lower ALFF or ReHo respectively. Representative diagrams of the differences of **D** ALFF and **E** ReHo in the 3D brain surfaces. Cg, cingulate cortex; Ent, entorhinal cortex; Hip, hippocampus; RSC; retrosplenial cortex. L, left; R, right

ALFF results indicated that MS groups exhibited the decreased ALFF values of the bilateral hippocampus and retrosplenial cortex versus SS group (Fig. 3A, B, D). The treatment with BHD significantly increased the ALFF values of the right hippocampus following ischemic stroke (Fig. 3A, B, D).

According to the ReHo data, the right hippocampus and entorhinal cortex of the MS group had abnormally higher ReHo values than those of the SS group (Fig. 3A, C, E). BHD treatment significantly decreased the ReHo value of the right hippocampus after cerebral ischemia (Fig. 3A, C, E).

### BHD facilitated the remodeling of cortical-hippocampal functional connectivity following cerebral ischemia

**Fig. 4:**
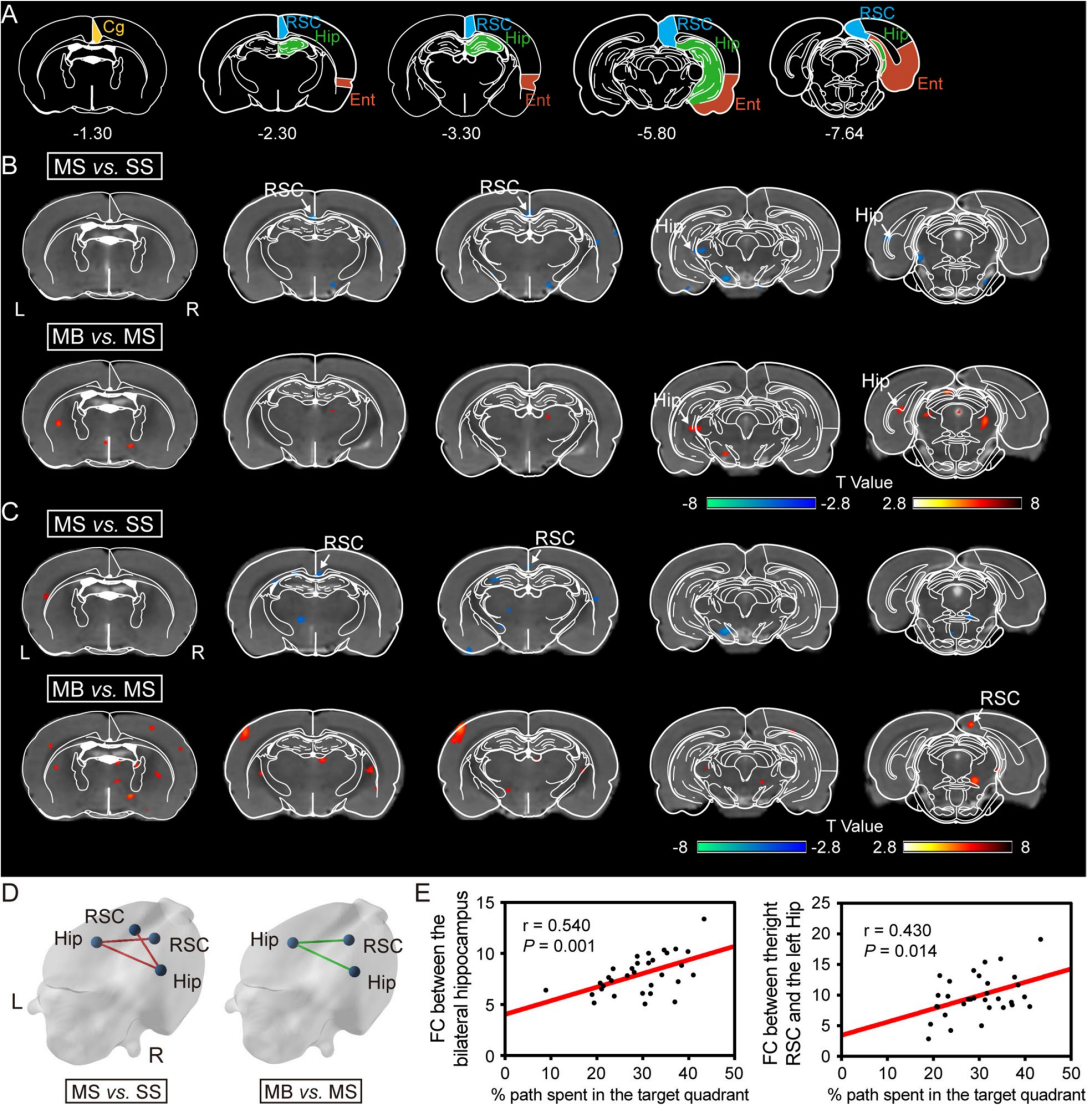
BHD facilitated the remodeling of the hippocampal-cortical functional connectivity after cerebral ischemia. **A** Schematic illustration in the Paxinos space. Distribution of significant differences of FC with the seed placed in the **B** right and **C** left hippocampus (FDR corrected, *P* < 0.005). The color overlay denotes the magnitude of group differences, with warm/cold hues representing higher/lower seed-based correlations respectively. **D** Representative figure of the differences of FC in the 3D brain surfaces. Red and green lines indicate decreased and increased connectivity, respectively. **E** Scatterplot of the correlation between the FC and the percentage of the path in the quadrant II in probe test. Cg, cingulate cortex; Ent, entorhinal cortex; Hip, hippocampus; RSC; retrosplenial cortex. L, left; R, right

With the seed placed in the right hippocampus, the MS group exhibited decreased FC between the right hippocampus and other areas, which involved the left hippocampus and retrosplenial cortex in comparison with SS group (Fig. 4A, B, D). BHD treatment raised FC value between the bilateral hippocampus after stroke (Fig. 4A, B, D).

When the seed was placed into the left hippocampus, the MS group showed decreased FC between the right retrosplenial cortex and the left hippocampus compared to the SS group. (Fig. 4A, C, D). Following BHD treatment, the FC value between the right retrosplenial cortex and the left hippocampus increased in MCAO rats (Fig. 4A, C, D).

Correlation results revealed the significant correlation between the inter-hemispheric FC (between the left hippocampus and the right retrosplenial cortex, and between the bilateral hippocampus) and the percentage of the path in the quadrant II in the probe test (Fig. 4E).

### BHD enhanced the post-stroke neurogenesis in the peri-infarct cortex

**Fig. 5:**
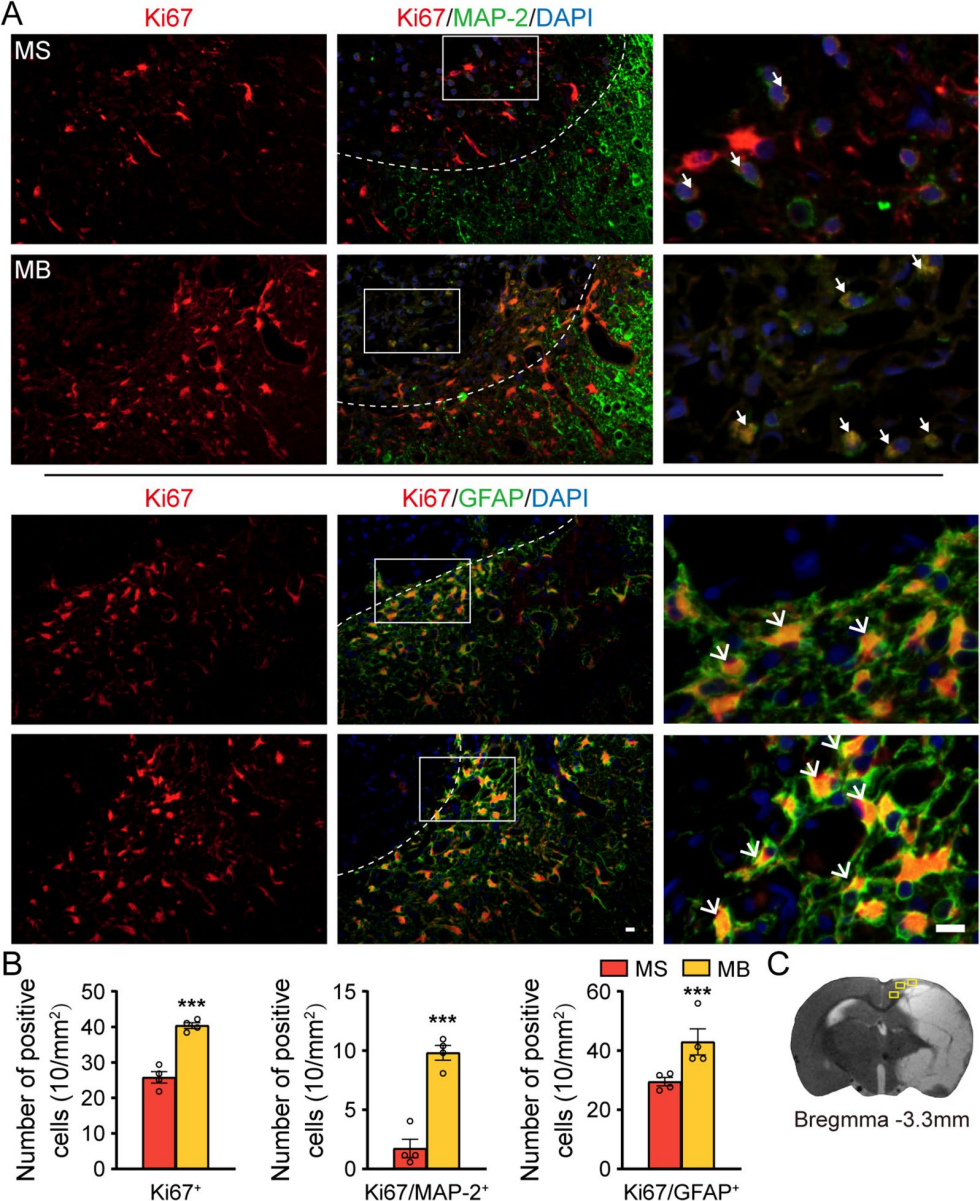
BHD enhanced the post-stroke neurogenesis of the peri-infarct cortex. **A** Representative images of immunofluorescence staining of Ki67/MAP-2^+^ and Ki67/GFAP^+^ from sections in the peri-infarct cortex. Scale bars = 10 μm. Co-located cells was labelled with white arrows. **B** Quantitative data of cell number of Ki67^+^, Ki67/MAP-2^+^ and Ki67/GFAP^+^ in the peri-infarct cortex. **C** Schematic figure of the peri-infarct cortex of rat brain diagram. Mean ± SEM, N = 4. ^*^P < 0.05,^**^P < 0.01, ^***^P < 0.001 vs. the MS group. R, right; L, left

Two-way ANOVA was used to identify the significant BHD × MCAO interaction on the number of Ki67^+^ , Ki67/MAP-2^+^ , and Ki67/GFAP^+^ cells in the peri-infarct cortex. Post hoc analysis showed that BHD treatment elevated the cell number of Ki67^+^, Ki67/MAP-2^+^ and Ki67/GFAP^+^ in the peri-infarct cortex following cerebral ischemia (Fig. 5).

### BHD regulated Shh signaling pathway in the cortex and hippocampus following ischemic stroke

**Fig. 6:**
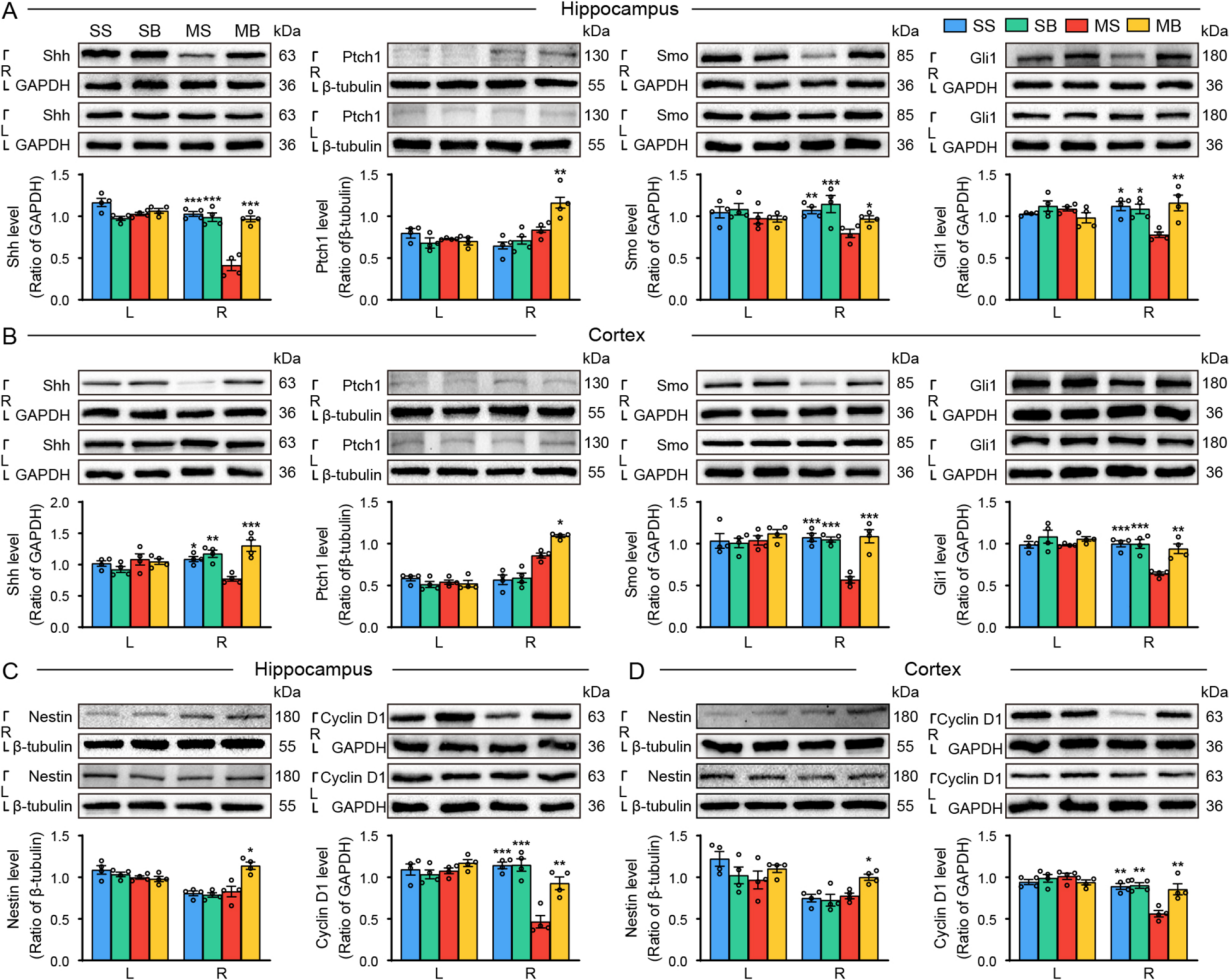
BHD regulated Shh signaling pathway in the hippocampus and cortex after cerebral ischemia. Representative diagram and quantitative results of Shh, Smo, Ptch1 and Gli1 in the (**A**) hippocampus and (**B**) cortex. Representative figure and quantitative results of Shh signaling downstream targets of the (**C**) hippocampus and (**D**) cortex. The level of the protein band was standardized with GAPDH or β-tubulin. Mean ± SEM, n = 4. ^*^P < 0.05, ^**^P < 0.01, ^***^P < 0.001 *vs.* the MS group. L, left; R, right

Our results showed the decreased levels of Shh, SMO and Gli1 in the right hippocampus and cortex, and elevated Ptch1 level in the right cortex (Fig. 6A, B). The treatment with BHD increased the levels of Shh, SMO, Ptch1 and Gli1 in the right hippocampus and cortex after stroke (Fig. 6A, B).

According to our findings, the right hippocampus and cortex had lower levels of Cyclin D1. (Fig. 6C, D). The treatment with BHD increased the expression of Cyclin D1 and Nestin in the right hippocampus and cortex (Fig. 6C, D).

## Discussion

The present study systematically evaluated the effects of cerebral ischemia on memory function in MCAO rats, as demonstrated through quantitative behavioral metrics in MWM. Ischemic injury manifested as significantly prolonged escape latency and reduced target quadrant (Quadrant II) dwell time, indicative of spatial memory impairment. Notably, BHD significantly attenuated these deficits, evidenced by reduced platform localization latency and enhanced quadrant-specific navigational preference. These quantifiable behavioral improvements, corroborating previous neuroprotective reports [32], suggest the therapeutic efficacy of BHD in ameliorating ischemia-induced memory dysfunction through mechanisms potentially involving hippocampal-cortical circuit modulation.

MRI, recognized as an objective and non-invasive technique, has been validated for its sensitivity to the tissue lesion following ischemia [40]. T2WI, a well-established modality for assessing structural alterations following ischemic stroke, revealed pronounced hyperintense signals in the right hemisphere, indicative of focal cerebral ischemia-induced lesions. Notably, therapeutic intervention with BHD demonstrated neuroprotective efficacy, as evidenced by amelioration of structural damage and enhanced preservation of cerebral tissue integrity.

Using diffusion tensor imaging (DTI), we evaluated BHD’s therapeutic effects on post-stroke microstructural damage in the hippocampus and cortex. DTI-derived fractional anisotropy (FA) values, sensitive to tissue integrity [41, 42], decreased significantly in right cortical and bilateral hippocampal regions after focal ischemia, reflecting microstructural disorganization. BHD treatment reversed these decreases, as evidenced by elevating FA values in affected areas, which was consistent with microstructural restoration [43]. These findings demonstrated the therapeutic potential of BHD to promote structural remodeling and neural recovery post-stroke.

Furthermore, we employed DTI to assess the impact of BHD treatment on the micro-structural lesions in the hippocampus and cortex after stroke. DTI-derived FA value is sensitive to the micro-structural lesion [41, 42]. A reduction in FA values reflects microstructural disorganization and tissue damage, whereas elevated FA values may indicate the restoration of microstructural integrity [43]. Herein, focal cerebral ischemia significantly reduced FA values in the right cortical regions and bilateral hippocampal formations. Following ischemic stroke, treatment with BHD significantly increased FA values in these affected regions, suggesting its potential role in promoting microstructural reorganization and repair within the hippocampus and cortex. These findings underscore the therapeutic potential of BHD in facilitating post-stroke neural remodeling.

BOLD-derived ALFF and ReHo analyses serve as robust, objective methodologies for evaluating regional spontaneous brain activity. Herein, focal cerebral ischemia attenuated spontaneous activity in the bilateral hippocampus and retrosplenial cortex. Moreover, ReHo analysis revealed increased regional homogeneity in the right hippocampus and entorhinal cortex following ischemic stroke. Of note, treatment with BHD activated the regional spontaneous activity in the right hippocampus. These findings align with prior evidence suggesting that enhanced neuronal activity facilitates neural circuit reorganization in rodent models of ischemic stroke, highlighting BHD's potential role in promoting post-stroke neuroplasticity [44, 45].

Coordination between the hippocampus and cortex serves a crucial role in the memory formation and storage [46]. Disruption of hippocampocortical connectivity resulted in significant impairments in memory function [22, 23]. The seed-based FC analysis in the current study indicated that unilateral ischemic stroke restricts the interhemispheric functional connectivity of the bilateral hippocampus. Moreover, cerebral ischemia decreased the interhemispheric functional connectivity between the retrosplenial cortex and the hippocampus. Notably, BHD not only strengthened the functional connectivity between the bilateral hippocampus but also enhanced the interhemispheric connection between the contralateral hippocampus and the ipsilateral retrosplenial cortex. Specifically, the correlation results showed that the BHD-induced increase of the percentage of the swimming path in the quadrant II was related to the strengthened inter-hemispheric hippocampal connectivity and hippocampal-cortical connectivity. Collectively, these results have identified the positive effect of BHD on the reformation of the hippocampal-cortical circuits, which was conducive to understanding the underlying mechanism of the improvement of the memory function.

Furthermore, we systematically evaluated the impact of BHD treatment on the neurogenesis within the peri-infarct regions after cerebral ischemia. Quantitative immunohistochemical analysis revealed the beneficial effect of BHD treatment on the neurogenesis in the peri-infarct area following ischemic stroke, as evidenced by the elevated cells number of Ki67^+^, Ki67/MAP-2^+^ and Ki67/GFAP^+^. These findings provide compelling cellular evidence for the therapeutic effect of BHD to enhance post-ischemic neurogenesis, suggesting its therapeutic potential in promoting neuronal and glial remodeling after stroke.

The Shh signaling pathway has been established as a critical regulator of hippocampal plasticity [29]. In present study, we investigated the expression profile of Shh signaling and downstream pathway in the hippocampal and cortical regions following cerebral ischemia. Herein, Ischemic injury significantly suppressed Shh, SMO, and Gli1 expression while upregulating Ptch1 levels in the right cortex and hippocampus. BHD treatment effectively reversed these alterations, promoting Shh pathway activation as evidenced by increased Shh, SMO, and Gli1 expression alongside decreased Ptch1 levels. Moreover, stroke-induced downregulation of downstream neurogenic markers (Cyclin D1 and Nestin) in the right hippocampus was ameliorated by BHD. These findings collectively demonstrate that BHD enhances post-stroke neurogenesis in cortical and hippocampal regions. Integrating functional connectivity data, we propose that BHD facilitates hippocampus-cortical circuit remodeling through modulation of Shh-mediated neurogenic signaling, thereby ameliorating ischemia-induced memory deficits. This mechanistic insight underscores the therapeutic potential of BHD in promoting functional recovery after cerebral ischemia.

The major Limitations of this study should be noted. This study utilized a two-way ANOVA to evaluate the interaction between MCAO and BHD, excluding multi-dose BHD groups. Limited to a 30-day post-ischemia time point, this study lacked temporal dynamics for comprehensive neuroplasticity insights. Future research will explore longitudinal and dose-dependent therapeutic effects of BHD to clarify neurorestorative mechanisms and optimize clinical protocols. In addition, future study will verify the Shh signaling in BHD-mediated neural circuit rewiring by blocking Shh in ischemic rats. Preliminary findings suggest BHD as a promising neuro-rehabilitative intervention, potentially mitigating post-stroke memory deficits through enhanced hippocampal-cortical plasticity, underscoring its therapeutic potential for cognitive recovery.

## Conclusions

Herein, *Buyang Huanwu* Decoction alleviated the micro-structural lesion and abnormal neuronal activity in the hippocampus and cortex, and promoted the enhancement of memory function after cerebral ischemia. Of note, BHD triggered the remodeling of the functional connectivity between the hippocampus and cortex after stroke. Indeed, BHD exerted pleiotropic effects on modulating the Sonic hedgehog signaling pathway in the hippocampus and cortex, which might underlie the mechanism of the remodeling of the hippocampal-cortical circuit after cerebral ischemia.

## Supplementary Information

Supplementary Material 1.

## Data Availability

The datasets throughout this study are available from the corresponding author upon reasonable request.

## Abbreviations

BHD: *Buyang Huanwu* Decoction
MCAO: Middle Cerebral Artery Occlusion
MRI: Magnetic Resonance Imaging
DTI: Diffusion tensor imaging
BOLD: Blood-oxygen-level-dependent
FA: Fraction Anisotropy
ALFF: Amplitude of Low Frequency Fluctuation
ReHo: Regional Homogeneity
FC: Functional Connectivity
Shh: Sonic Hedgehog
Ptch1: Patched
Smo: Smoothened
