# Supplementary material for: Buyang Huanwu Decoction enhances hippocampal-cortical connectivity remodeling via sonic hedgehog signaling to ameliorate memory dysfunction in cerebral ischemic rats
Source: Chin Med. 2025 Sep 19;20:144. doi: 10.1186/s13020-025-01122-0 (PMC12447606; doi:10.1186/s13020-025-01122-0)
Supplement: Supplementary file 1 — Supplementary Material 1. [file 13020_2025_1122_MOESM1_ESM.docx]

Supplementary material for:

***Buyang Huanwu* Decoction activated the neuronal plasticity via regulating Sonic hedgehog signaling to improve the memory dysfunction following cerebral ischemia: A fMRI-based FC analysis in rats**

**1. The parameters of MRI sequence**

| Imaging | Sequence | Parameters |
| --- | --- | --- |
| T2WI | a fast spin-echo pulse sequence | TR = 4400 ms, TE = 45 ms, Matrix size = 256×256, flip angle = 180°, Field of View = 3.3×3.3 cm, 38 slices, slice thickness = 0.7 mm, slice gap = 0, Scan time = 4 min. |
| DTI | an axial single-shot spin echo-planar imaging sequence | TR/TE = 6300/25 ms, 30 diffusion encoding directions, two b values = 0 and 1000 s/mm2, Matrix size = 128×128, Flip angle = 90°, Field of View = 3.5×3.5 cm2, Number of excitation = 1, Scan time = 10 min. |
| BOLD | an EPI-SE-FOVsat sequence | TR = 2000 ms, TE = 11 ms, Matrix size = 80×64, flip angle = 90°, Field of View = 2.5×2.0 cm, 38 slices, slice thickness = 0.7 mm, slice gap = 0, Scan time = 8 min. |
